# Nucleotide sequence and analysis of pRC12 and pRC18, two theta-replicating plasmids harbored by *Lactobacillus curvatus* CRL 705

**DOI:** 10.1371/journal.pone.0230857

**Published:** 2020-04-02

**Authors:** Lucrecia C. Terán, Sergio A. Cuozzo, María C. Aristimuño Ficoseco, Silvina Fadda, Stéphane Chaillou, Marie-Christine Champomier-Vergès, Monique Zagorec, Elvira M. Hébert, Raúl R. Raya

**Affiliations:** 1 Centro de Referencia para Lactobacilos (CERELA)-CONICET, Tucumán, Argentina; 2 Planta Piloto de Procesos Industriales Microbiológicos (PROIMI)-CONICET, Tucumán, Argentina; 3 Université Paris-Saclay, INRAE, AgroParisTech, Micalis Institute, Jouy-en-Josas, France; 4 INRAE, UMR1014 Secalim, Oniris, Nantes, France; All India Institute of Medical Sciences, Bhopal, INDIA

## Abstract

The nucleotide sequences of plasmids pRC12 (12,342 bp; GC 43.99%) and pRC18 (18,664 bp; GC 34.33%), harbored by the bacteriocin-producer *Lactobacillus curvatus* CRL 705, were determined and analyzed. Plasmids pRC12 and pRC18 share a region with high DNA identity (> 83% identity between RepA, a Type II toxin-antitoxin system and a tyrosine integrase genes) and are stably maintained in their natural host *L*. *curvatus* CRL 705. Both plasmids are low copy number and belong to the theta-type replicating group. While pRC12 is a pUCL287-like plasmid that possesses iterons and the *repA* and *repB* genes for replication, pRC18 harbors a 168 amino acid replication protein affiliated to RepB, which was named RepB’. Plasmid pRC18 also possesses a pUCL287-like *repA* gene but it was disrupted by an 11 kb insertion element that contains RepB’, several transposases/IS elements, and the lactocin Lac705 operon. An *Escherichia coli* / *Lactobacillus* shuttle vector, named plasmid p3B1, carrying the pRC18 replicon (*i*.*e*. *repB’* and replication origin), a chloramphenicol resistance gene and a pBluescript backbone, was constructed and used to define the host range of RepB’. Chloramphenicol-resistant transformants were obtained after electroporation of *Lactobacillus plantarum* CRL 691, *Lactobacillus sakei* 23K and a plasmid-cured derivative of *L*. *curvatus* CRL 705, but not of *L*. *curvatus* DSM 20019 or *Lactococcus lactis* NZ9000. Depending on the host, transformation efficiency ranged from 10^2^ to 10^7^ per μg of DNA; in the new hosts, the plasmid was relatively stable as 29–53% of recombinants kept it after cell growth for 100 generations in the absence of selective pressure. Plasmid p3B1 could therefore be used for cloning and functional studies in several *Lactobacillus* species.

## Introduction

The genus *Lactobacillus* has a great importance for the food industry since many lactobacilli are used as starter cultures for manufacturing various fermented foods, beverages, and feed products [1]. In addition, some *Lactobacillus* strains are increasingly marketed as health-promoting probiotic bacteria or as putative protective cultures for the biopreservation of food [2–4].

*Lactobacillus* strains harbor plasmids, small DNA molecules that can replicate independently of the chromosomal DNA. Plasmids were detected in 41% of 213 sequenced genomes of *Lactobacillus* and associated lactic acid bacteria (LAB) [5]. Although many of these plasmids are cryptic and their role in bacterial physiology is not known, some are responsible for various phenotypes, such as exopolysaccharide synthesis, bacteriocin production, carbohydrate metabolism, or proteolytic activity [6].

Two main mechanisms of replication have been reported in *Lactobacillus* plasmids: rolling circle and theta types [7]. Plasmids that replicate via the latter mechanism are usually more stable. This characteristic is of interest for the construction of vectors carrying gene expression systems. However, the theta-type plasmids have usually a narrower host range than plasmids that replicate via rolling circle [8]. Accordingly, knowledge about host range, copy number and stability of plasmids is especially important for developing genetic tools for the study of LAB. In general, most *Lactobacillus* strains are difficult to transform and lactobacilli plasmids are the best choice for constructing shuttle vectors. Several *Lactobacillus* plasmids have been used as a backbone for developing cloning vectors, such as pRV500, pMC11, pYC2, and pSIP [9–13]. Some features of the vectors (size, replication system, etc.) as well as the electrical parameters used for electroporation influence transformation frequency [14].

*Lactobacillus curvatus* is a facultative heterofermentative LAB traditionally associated with fermented meat products, vacuum-packaged refrigerated meat, poultry, and fish products [15]. This species is ubiquitous as it was also isolated from other ecological niches, such as fermented vegetables and dairy products [16–18]. To date, the sequences of 19 *L*. *curvatus* plasmids, with sizes ranging from 2 kb to 61 kb, have been uploaded in the NCBI database. While some strains harbor only one plasmid [i.e., strain FLEC03 hosts plasmid pLCUFL03 (47 kb; NZ_LT841332.1) [19] and strain KG6 contains plasmid pKG06 (17 kb; NZ_CP022476)], other strains have up to five plasmids each (i.e., strains TMW1.1928 and NFH-Km12) [20].

*L*. *curvatus* CRL 705 (previously described as *Lactobacillus casei* CRL 705) was isolated from a traditional Argentinian sausage [21]. It produces two types of bacteriocins, lactocin Lac705 and AL705 (anti-listeria activity), and harbors two plasmids, pRC12 and pRC18. The production of lactocin Lac705 has been associated with the plasmid pRC18 [22, 23] and is a quite unique bacteriocin among LAB, as the active peptides Lac705β and Lac705α do not show any homology with any protein deposited in data bases (http://pfam.xfam.org/family/PF08109). Strain CRL 705 is a model protective culture that can prevent the growth of food spoilage bacteria and improve storage life of refrigerated vacuum-packaged beef without affecting its sensory and structural characteristics [21]. The objective of this study was to determine and analyze the sequences of pRC12 and pRC18, the two plasmids harbored by *L*. *curvatus* CRL 705 that share regions with high identity, as the plasmidome of this species is not yet well understood. Besides, the replication protein RepB’ of pRC18, a replicase that has not been previously studied and may represent a potential tool for creating new cloning vectors, was characterized.

## Materials and methods

### Bacterial strains, plasmids and growth conditions

Bacterial strains and plasmids used in this study are listed in Table 1. *Lactobacillus* strains were grown in MRS at 30°C [24]. *Lactococcus lactis* was grown at 30°C in M17 medium [25] with 0.5% glucose. *Escherichia coli* DH10B was grown in Luria Bertani broth [26] with agitation at 37°C. When necessary, 100 **μ**g/ml of ampicillin for *E*. *coli*, and 5 **μ**g/ml of chloramphenicol or 1 **μ**g/mL of erythromycin for LAB strains were added to the media.

**Table 1:** Strains and plasmids used in this study.

Strain or Plasmid	Features	Source/reference
*Strains*		
*Escherichia coli*	DH10B	[27]
*L*. *curvatus* CRL 705	Wild-type strain, natural host of pRC12 and pRC18	[21]
*L*. *curvatus* CRL 1890	Plasmid-cured derivative of CRL 705.	This study
*L*. *curvatus* DSM20019	Wild-type strain, used for transformation tests	[28]
*L*. *plantarum* CRL 691	Wild-type strain, used for transformation tests	[21]
*L*. *sakei* 23K	Plasmid cured strain, used for transformation tests	[14]
*Lactococcus lactis* NZ9000	Wild-type strain, used for transformation tests	[29]
*Plasmids*		
pRC18	Wild-type plasmid from *L*. *curvatus* CRL 705	[22][23]
pRC12	Wild-type plasmid from *L*. *curvatus* CRL 705	[23]
pBlueScript II SK (+)	pBS vector, AmpR	Stratagene
p3B1	pBS derivative with RepB from pRC18, AmpR, CmR	This study
pRV300	pBS derivative with *ermAM*, AmpR, EryR	[30]
pC194	CmR	[31]
pRV566	pRV300 derivative with RepA from pRV500, AmpR, EryR	[9]
pRA1TA	p3B1 derivative with toxin-antitoxin system from pRC18	This study

### Plasmid manipulation and construction

Plasmid DNA was extracted using standard procedures for LAB [32] and *E*. *coli* [33]. p3B1 plasmid was constructed by ligating the *Hpa*I-*Eco*RI (3.47 kb; coordinates 10334–13806) fragment of pRC18 into the *Hin*dIII (blunted with Klenow plus dNTPs)- *Eco*RI sites of plasmid pBlueScript II SK (+). Then, the chloramphenicol gene from plasmid pC194 (a 1,035 bp *Hpa*II-*Mbo*I fragment, nt 973–2,006 of pC194, blunted with Klenow and dNTPs) was cloned at the *Sma*I restriction site of the recombinant pBlueScript-pRC18 derivative plasmid. Plasmid pRA1TA (7,139 bp) was constructed as follows: toxin and antitoxin genes were amplified by PCR using plasmid pRC18 DNA as a template, with primers RRta Fw and RRta Rv (Table 2) and *Pfu* DNA polymerase (Promega, Buenos Aires, Argentina). The amplified 983-bp fragment was treated with restriction endonuclease *Eco*RI, ligated to plasmid p3B1 (digested with *Eco*RI and *Eco*RV), and used to transform *E*. *coli*. Transformants were selected with ampicillin and the recombinant plasmid pRA1TA was confirmed by DNA sequencing.

**Table 2:** Primers used for plasmid copy number determination by quantitative PCR.

Name	Sequence 5`→3`	Melting Temperature (°C)	Fragment length (bp)
*rpoD* Fw	CGGTTGGTGCAGTCATATCT	62	151
*rpoD* Rv	GCGCTACAAGCTGATGAAATC		
pRC18 Fw	CATGCGGGATCAAGGGAATAA	62	156
pRC18 Rv	CTCTTACTTCTACGGCCAAACC		
pRC12 Fw	CTGAGTTAGGCCAGCTTCTT	62	157
pRC12 Rv	CGAACTGAATGGGAGGATAGTC		
RRta Fw	GGGGAATTCTCCCCACACATTAGGGTCT	76	983
RRta Rv	GGGAGATCTCGCAGCAGTGTCGCTTTGCC	82	

### Competent cells and electroporation

*E*. *coli* DH10B competent cells were prepared and transformed after heat shock at 42°C for 2 min [34]. Preparation of *Lactococcus lactis* competent cells and transformation were done as previously described [35]. *Lactobacillus* competent cells were prepared following the procedures described by Berthier et al [14]. Briefly, cells were grown in MRS broth for about 6 h until an optical density between 0.4–0.6. Then, the cells were washed twice with MgCl_2_ 10 mM and then with a storage solution of sucrose 0.5 M containing 10% glycerol, in which they were suspended and stored at -80°C. Electrotransformation of *L*. *curvatus* CRL 705 cells was optimized using the plasmid pRV566 and varying systematically the electroporation parameters (voltage: 1.8 kV, 2.0 kV and 2.5 kV; resistance: 200 Ω, 600 Ω, 800 Ω and 1000 Ω. The optimized parameters were then used to electroporate *L*. *curvatus* and *L*. *plantarum* strains, *L*. *sakei* 23K cells were electroporated using 1.8 kV, 600 Ω and 25 **μ**F as previously described [14].

### Determination of relative plasmid copy number

The relative plasmid copy number was determined using quantitative real time PCR (qPCR), following the procedures described by Lee and coworkers [36]. As a reference, a 151 bp fragment of the *rpoD* gene was used, which has been identified as a single copy in the chromosome. Two other pairs of primers were used to amplify same-sized fragments of pRC18 and pRC12 plasmids (Table 2). Primers for qPCR were designed using IDT Primer Quest (https://www.idtdna.com/Primerquest/Home/Index) and synthetized by Sigma Aldrich (Buenos Aires, Argentina). The copy number was calculated according to the formula: N_relative_ = (1+E) –^ΔCT^ [36], where E is the PCR amplification efficiency of the target and reference genes, and ΔCT stands for the difference in the threshold cycle number between the reference and the target genes. The experiment was carried out using three replicates for both, biological and technical repetitions. The results were expressed in average values and the standard errors were calculated from the data with all the repetitions. The plasmid copy number was then classified as low, medium or high when the copies of plasmids per chromosome were 1–10 copies, 11–20 copies or more than 20 copies, respectively.

### Host range and stability tests

The host range of the novel replicase (RepB’) found in pRC18 was defined using different LAB (Table 1) as transformations hosts with p3B1 vector. The presence of p3B1 was confirmed by plasmid DNA extraction and PCR, using primers 5`-CGGGAAACCGGTGAAATCTA-3` and 5`-CCTACTCCGCAATCGCTAAA-3`, under the following conditions: the first step for denaturalization at 94°C during 5 min, then 35 cycles for extension including 1 min at 94°C, 2 min at 58°C and 1 min at 72°C followed by a final extension of 5 min at 72°C.

The segregational stability of p3B1 was assayed following the protocol described by Alpert et al [9] with slight modifications: strains were reinoculated in fresh MRS medium every 24 h, using appropriate dilutions, until approximately 100 generations. Cells were then plated on agar media with or without antibiotic and incubated at 30°C. Colonies forming units (CFU) per mL were determined by biological and technical triplicates, the average values and the standard error were calculated.

### Plasmid DNA sequencing and sequence analysis

DNA sequencing was performed as previously described by our team [23]. Coding sequences (CDSs) were predicted by Glimmer (https://www.ncbi.nlm.nih.gov/genomes/MICROBES/glimmer_3.cgi). Functional annotation of CDSs encoded on plasmid pRC12 was performed considering the identities using BLAST (http://www.ncbi.nlm.nih.gov/BLAST) and InterProScan (http://www.ebi.ac.uk/interpro/) and other tools available for sequence analysis in Microscope Platform [37]. GC content was calculated using http://www.endmemo.com/bio/gc.php. Prediction of tRNA was conducted with tRNAscan-SE (http://lowelab.ucsc.edu/tRNAscan-SE/). Plasmid maps of pRC12 and pRC18 were generated using SnapGene software (from GSL Biotech; snapgene.com). Comparative analysis between pRC18 and pRC12 was performed with Easy Fig [38] and comparison maps of *L*. *curvatus* plasmids were drawn using Gview Server [39] and Circoletto [40].

### Nucleotide sequence accession number

For comparative analyses, the following *L*. *curvatus* plasmids were used: pLCUFL03 (NZ_LT841332.1), pKG6 (NZ_CP022476.1), pIRG2 (NZ_CP025475.1), pLDW-27 (CP026117.1), pZJUNIT8_1 (NZ_CP029967.1), pZJUNIT8_2 (NZ_CP029968.1), pZJUNIT8_3 (NZ_CP029969.1), pZJUNIT8_4 (NZ_CP029970.1), pNFH-Km12_1 (AP018700.1), pNFH-Km12_2 (AP018701.1), pNFH-Km12_3 (AP018702.1), pNFH-Km12_4 (AP018703.1), pNFH-Km12_5 (AP018704.1), p-1.1928_1 (NZ_CP031004.1), p-1.1928_2 (NZ_CP031005.1), p-1.1928_3 (NZ_CP031006.1), p-1.1928_4 (NZ_CP031007.1) and p-1.1928_5 (NZ_CP031008.1). Also, plasmid pUCL287 from *Tetragenococcus halophilus* ATCC 33315 (X75607.1) and *L*. *sakei* CECT 9267 (NZ_OKRC01000015) were used. The nucleotide sequences of plasmids pRC12 and pRC18 were deposited in the NCBI under the accession numbers MF383377 and AF200347, respectively.

## Results

The aim of this study is the analysis and characterization of plasmids pRC12 and pRC18 detected in the draft genome sequence of *L*. *curvatus* CRL 705 [23]. While the nucleotide sequence of pRC12 was determined as a single contig (23-fold coverage) by using a whole-genome shotgun (WGS) strategy, the genome sequence of pRC18 was determined by a primer-walking strategy and confirmed by WGS [23]. CDSs of more than 70 nucleotides in length were identified and annotated. The presence of tRNA was not detected. Both plasmids belong to the theta replication group and the relative plasmid copy number of pRC18 and pRC12, as determined by quantitative real time PCR (qPCR), was 6.41 ± 1.20 and 3.60 ± 0.57 copies per cell, respectively. Thus, pRC12 and pRC18 are low-copy number plasmids.

### Sequence analysis of pRC12

**Fig 1:**
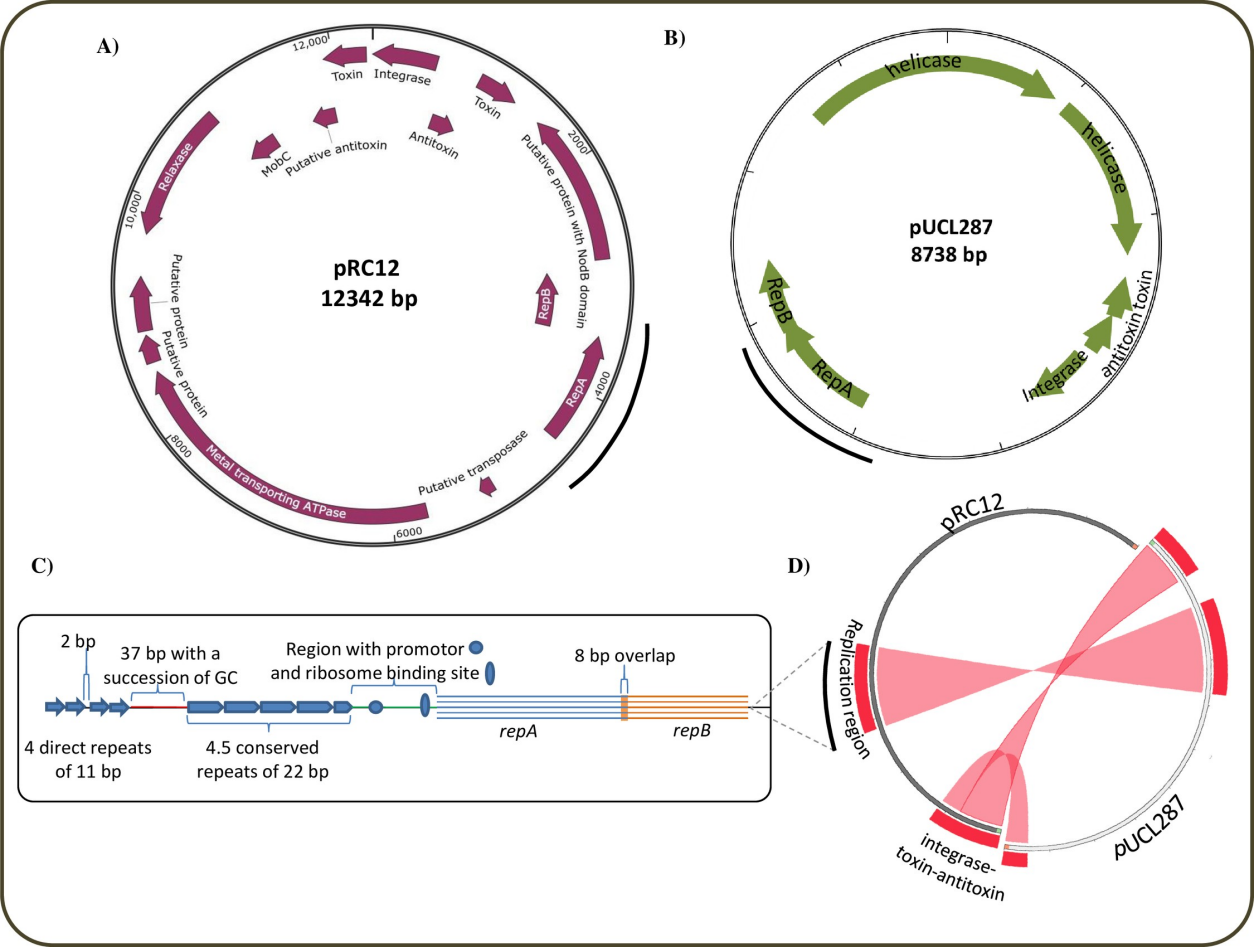
Structure and organization of pRC12. A) Physical and genetic map of plasmid pRC12 from *L*. *curvatus* CRL 705. Orientation of deduced CDSs are shown by purple arrows. B) Genetic map of plasmid pUCL287 (X75607.1) from *Tetragenococcus halophilus* ATCC33315. C) Structural organization of the replicative region of plasmids pRC12 and pUCL287. D) Comparison between plasmids pRC12 from *L*. *curvatus* CRL 705 and pUCL287 from *Tetragenococcus halophilus* ATCC33315; both plasmids share high identity of the replication region (*ori*-*repA*) and the integrase and toxin-antitoxin system.

The DNA sequence of plasmid pRC12 consists of 12,342 bp with 43.99% GC content. The average length of CDSs is about 638 bp and the intergenic average length is 249 bp. Fourteen putative proteins encoding CDSs (designated ORF1 to ORF14) were identified (Fig 1A, Table 3). ORF 1 encodes a site-specific tyrosine recombinase (integrase) that belongs to the conserved protein domain family INT_C_like_3 and it is also present in different strains of the genus *Lactobacillus* (100% identity). Downstream from ORF1 and in the opposite orientation, ORF 2 and ORF 3 encode an antitoxin of the Phd/YefM family and a RelE-like toxin, respectively, a type II toxin-antitoxin stability system. The protein encoded by ORF 4 was predicted to be a hydrolase/deacetylase, and contains a NodB catalytic domain of the carbohydrate esterase 4 superfamily. Two CDSs involved in the replication process were identified: ORF 5 and ORF 6 correspond to RepB and RepA, respectively, two proteins involved in the replication of theta-type plasmids found in other LAB. In particular, the genetic organization of the replication system (*ori*-*repA*) of pRC12 plasmid is consistent with that of the well characterized plasmid pUCL287 from *Tetragenococcus halophilus* ATCC33315 [41, 42] (Fig 1B–1D). Indeed, ORF 6 (*repA*) encodes a 311 amino acids (aa) protein 100% identical to RepA from *L*. *sakei* subsp. *carnosus* DSM15831 (KRL70440.1) while ORF 5 (*repB*) encodes a 192 aa protein, with 99% identity with the replication protein RepB found in *L*. *curvatus* KG6 (ASN62919.1). *repA* and *repB* overlap by 8 bp as in pUCL287 (Fig 1D), the untranslated region upstream from *repA* consists of four 11 bp direct repeats, with two of them being separated from the other two by 2 bp. These four repeats are then separated by 37 bp from a 22 bp motif (iterons) repeated 4.5 times (Fig 1C and S1 Fig). These iterons may constitute the replication origin of pRC12. Between these repeats and the *repA* start codon, the promoter and ribosome binding sites (Fig 1C) are located, as reported in [41, 42]. ORF 7 is a remnant gene that consists of a putative transposase. ORF 8 encodes a putative metal-transporting ATPase, while ORF 9 and ORF 10 encode conserved proteins of unknown function.

**Table 3:** Functions of pRC12 CDSs and similarities with proteins in the GenBank database.

ORF	Begin	End	Frame	AA	Proposed function	AA Identity (%)^a^
**1**	1	558	-1	195	Integrase	100% integrase family protein [*L*. *capillatus* DSM19910] **KRL03353.1**
**2**	667	945	+1	92	Phd/YefM Antitoxin family of toxin-antitoxin stability system Type II	93% prevent-host-death family protein [*L*. *brevis* WK12] **WP_042253922.1**
**3**	945	1301	+3	118	Toxin, RelE/StbE family protein of toxin-antitoxin stability system	99% addiction module toxin RelE [*L*. *lindneri* TMW 1.481 plasmid pL1481-4] **ANZ59883.1**
**4**	1552	2865	-1	424	Putative protein of unknown function with NodB catalytic domain of the carbohydrate esterase 4 superfamily	99% multispecies: lipoprotein [*Lactobacillus*] **WP_044011365.1**
**5**	2952	3530	-3	192	RepB replication protein of pUCL287-like	99% DUF536 domain-containing protein (plasmid) [*L*. *curvatus*] **ASN62919.1**
**6**	3523	4458	-1	311	RepA replication initiation protein of pUCL287-like	100% replication initiation protein [*L*. *sakei* subsp. *carnosus* DSM15831] **KRL70440.1**
**7**	5101	5256	+1	51	Putative transposase (fragment)	83% transposase [*Pediococcus acidilactici* D3] **EOA08716.1**
**8**	5706	8510	+3	934	Metal transporting ATPase	100% cation-transporting ATPase, E1-E2 family (plasmid) [*L*. *sakei*] **SON74352.1**
**9**	8594	8833	+1	79	Putative conserved protein of unknown function	100% hypothetical protein, partial [*L*. *paracasei* subsp. *paracasei* Lpp221] **AGL65813.2**
**10**	8869	9336	+1	155	Putative conserved protein of unknown function	100% multispecies: hypothetical protein [*Lactobacillus*] **WP_016511518**
**11**	9695	10864	-3	389	Relaxase/mobilization protein domain	100% relaxase/mobilization nuclease domain protein (plasmid) [*L*. *sakei* MFPB19] **SON74348.1**
**12**	10846	11199	-1	117	Mobilization protein MobC	100% mobilization protein MobC [*Pediococcus damnosus*] **KRN47316.1**
**13**	11664	11933	-2	89	Antitoxin of toxin-antitoxin system; Cro/C1-type HTH domain; COG1396	100% DNA-binding helix-turn-helix protein [*L*. *hilgardii* DSM20176] **EEI23308.1**
**14**	11920	12291	-1	123	Toxin RelE family of toxin-antitoxin system Type II, HigB-like; COG4679	100% multispecies: type II toxin-antitoxin system RelE/ParE family toxin [Bacteria] **WP_001748275.1**

^a^ percentage of identity determined by BLASTp (NCBI database) followed by protein name, bacterial name (in brackets) and the accession number.

pRC12 also harbors two CDSs related to DNA mobilization: ORF 11, which encodes a protein highly similar to the relaxase/mobilization domain found in the plasmid content of *L*. *sakei* MFPB19 (SON74348.1), and the protein encoded by ORF 12, which is homologous to MobC of *Pediococcus damnosus* (KRN47316.1). Finally, a second type II toxin-antitoxin system is harbored by pRC12, divergent from that encoded by ORF2 and ORF3. ORF 13 and ORF 14 encode a putative antitoxin of the lambda repressor-like, Cro/C1-type HTH domain, DNA-binding domain superfamily (COGnitor: COG1396 group) and a HigB-like toxin (COGnitor: COG4679 family), respectively.

### Sequence analysis of pRC18

**Fig 2:**
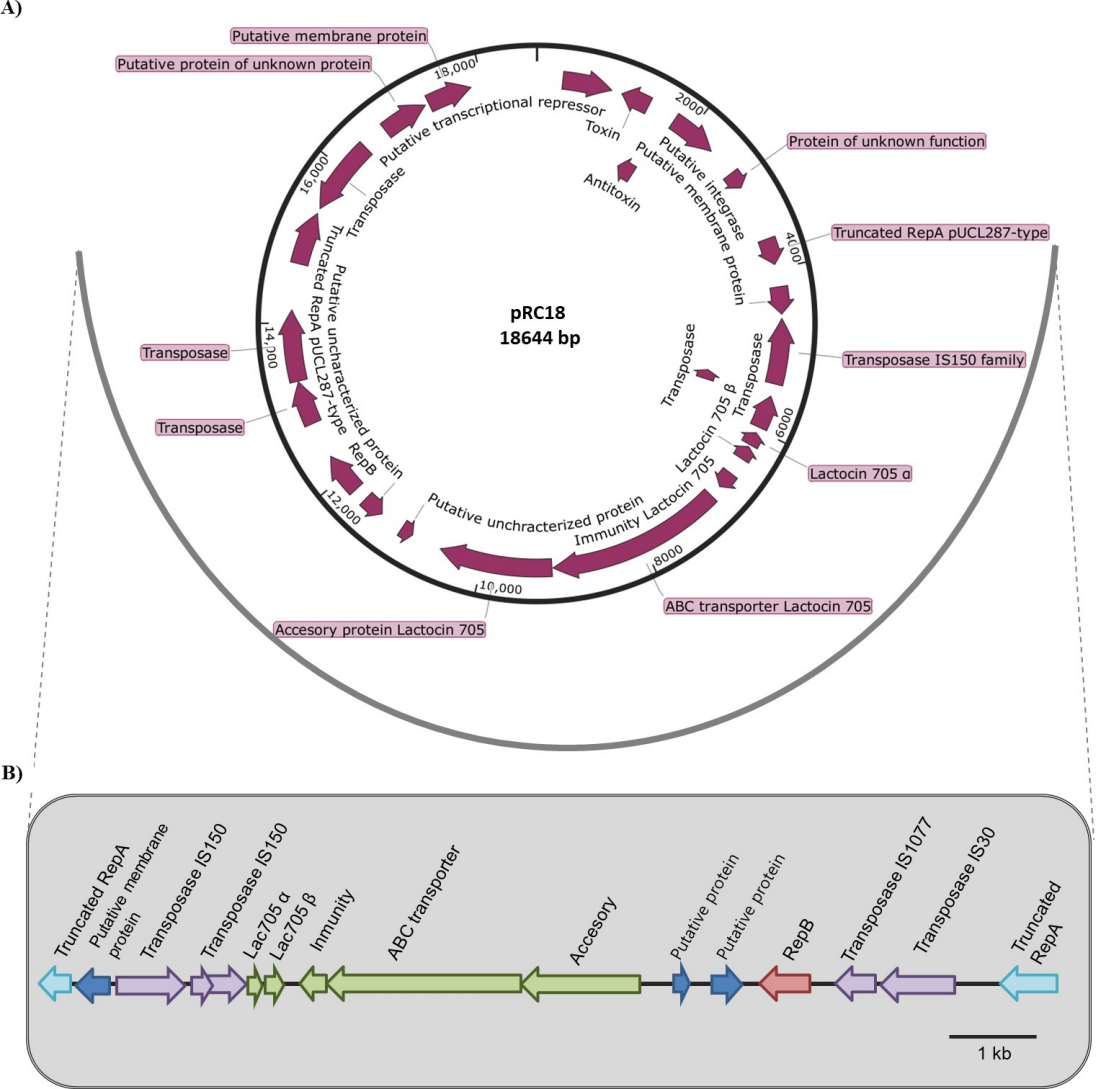
Structure and organization of pRC18. A) Physical and genetic map of the plasmid pRC18 from *L*. *curvatus* CRL 705. Deduced CDSs are shown by purple arrows indicating their orientation. B) Focus on an 11 kb insertion disrupting *repA* showing the presence of transposases, and the lactocin 705 operon.

The nucleotide sequence of plasmid pRC18 consists of 18,664 bp, with a GC content of 34.33%. Twenty-four CDSs were identified in this plasmid, with an average CDS length of 557 bp and an average intergenic length of 213 bp (Fig 2A, Table 4); the protein coding density is 65.32%.

**Table 4:** Functions of pRC18 CDSs and similarities of their encoded proteins present in the GenBank database.

ORF	Begin	End	Frame	AA	Proposed function	AA identity (%)^a^
**1**	319	927	+1	202	Putative HTH and XRE transcriptional repressor family protein	100% transcriptional regulator (plasmid) [*L*. *paracollinoides* TMW 1.1994] **ANZ65517.1**
**2**	1055	1411	-1	118	Toxin of toxin-antitoxin stability system, RelE/StbE family protein	100% addiction module toxin RelE (plasmid) [*L*. *lindneri*] **ANZ59883.1**
**3**	1411	1689	-2	92	Phd/YefM Antitoxin family of toxin-antitoxin stability system	100% prevent-host-death family protein [*L*. *brevis*] **WP_042253922.1**
**4**	1766	2353	+2	195	Putative integrase	99% integrase [*L*. *brevis* 47f] **KLE28650.1**
**5**	2731	2925	+1	64	Protein of unknown function	100% hypothetical protein [*L*. *plantarum* 4_3] **ETF10394.1**
**6**	3622	3945	+1	107	Truncated replication protein RepA. pUCL287-type replication system	100% initiator Replication protein [*L*. *sakei*] **SPE23715.1**
**7**	4221	4553	+3	110	Putative membrane protein of unknown function	100% hypothetical protein LAS9267_02046 [*L*. *sakei*] **SPE23716.1**
**8**	4606	5406	-2	266	Transposase (IS150 family)	98% IS3 family transposase [*L*. *curvatus*] **ASN62826.1**
**9**	5448	5606	-3	52	Transposase (IS150 family)	98% hypothetical protein NFHkm12_00970 [*L*. *curvatus*] **BBE25271.1**
**10**	5661	5965	-1	134	Transposase (frameshifted)	99% hypothetical protein NFHkm12_11490 [*L*. *curvatus*] **BBE26323.1**
**11**	6039	6203	-3	54	Lactocin 705 alpha	100% bacteriocin lactocin-705 [*Lactobacillus sakei*] **SPE23718.1**
**12**	6230	6400	-1	56	Lactocin 705 beta	100% COMC family protein [*L*. *sakei*] **SPE23719.1**
**13**	6605	6883	+2	92	Immunity protein Lactocin 705 system	100% hypothetical protein LAS9267_02050 [*L*. *sakei*] **SPE23720.1**
**14**	6979	9135	+1	718	ABC transporter Lactocin 705 system	100% Lactococcin-G-processing and transport ATP-binding protein LagD [*L*. *sakei*] **SPE23721.1**
**15**	9148	10533	+1	461	Bacteriocin secretion accessory protein, Lactocin 705 system	99% Lactococcin A secretion protein LcnD [*L*. *sakei*] **SPE23722.1**
**16**	10906	11061	-2	51	Putative uncharacterized protein	-
**17**	11355	11627	-3	90	Putative uncharacterized protein	-
**18**	11790	12296	+3	168	RepB’	100% multispecies: DUF536 domain-containing protein [*Lactobacillus*] **WP_011005830.1**
**19**	12749	13285	+2	178	Transposase	100% multispecies: transposase [Lactobacillaceae] **WP_011005829.1**
**20**	13294	14163	+1	289	Transposase (IS150 family)	100% transposase [*L*. *collinoides*] **KZL35624.1**
**21**	14729	15382	+2	217	Truncated replication protein RepA. pUCL287-type replication system	100% initiator replication protein [*L*. *sakei*] **SPE23725.1**
**22**	15447	16367	-3	306	Transposase (IS30 family)	100% putative transposase IS1070 [*L*. *salivarius* GJ-24] **EGM49769.1**
**23**	16690	17265	+1	191	Putative protein of unknown function	99% hypothetical protein LAS9267_02056 [*L*. *sakei*] **SPE23726.1**
**24**	17311	17859	+1	549	Putative membrane protein of unknown function	99% hypothetical protein [*L*. *sakei*] **WP_105300171.1**

^a^ percentage of identity determined by BLASTp (NCBI database) followed by protein name, bacterial name (in brackets) and the accession number.

ORF 1 encodes a 202 aa protein 95% identical to Xre family transcriptional regulator of *Lactobacillus paracollinoides* TMW1.1994. As reported above for pRC12, an *int-antitoxin-toxin* operon is also found in plasmid pRC18. ORF 2 and ORF 3 encode a toxin of the RelE-like and an antitoxin of the Phd/YefM family, respectively. ORF 4 encodes a 195 aa integrase of the INT_C_like_3 DNA breaking-rejoining enzyme superfamily.

pRC18 plasmid has a pUCL287-like replication initiator protein RepA which is disrupted in two parts (ORF 6 and ORF 21, the 5’ and 3’ fragments of *repA*, respectively) by an 11 kb insertion (Fig 2B). ORF 6 shows 92% identity with the N-terminus of the RepA protein of plasmid pPECL-7 from *Pediococcus damnosus* LMG 28219 and ORF 21 shows 99% identity with the C-terminus of the RepA protein found in *L*. *paracollinoides* DSM 15502. The 11 kb fragment encompasses a transposase-like structure. ORF 8, ORF 9, ORF 10 (frameshifted) and ORF 20 are related to transposases of the IS150 family, whereas ORF 19 and ORF 22 are transposases of the IS1077 and IS30 families, respectively. Moreover, the 11 kb insertion contains the lactocin Lac705 operon (ORFs 11 to 15), a class IIb bacteriocin that relies on the complementary action of two peptides, Lac705α and Lac705β [22, 43]. ORF 18 encodes a putative replicase that we named RepB’ and is described below. The 11 kb fragment is highly similar to a contig found in the recently sequenced *L*. *sakei* CECT 9267 (NZ_OKRC01000015) (S2 Fig). Finally, ORFs 16–17 and ORFs 23–24 did not show significant homology to any protein available in GenBank and Phyre 2 databases.

### Comparative nucleotide sequence analysis of pRC18 against pRC12 and other plasmids harbored by *L*. *curvatus* strains

**Fig 3:**
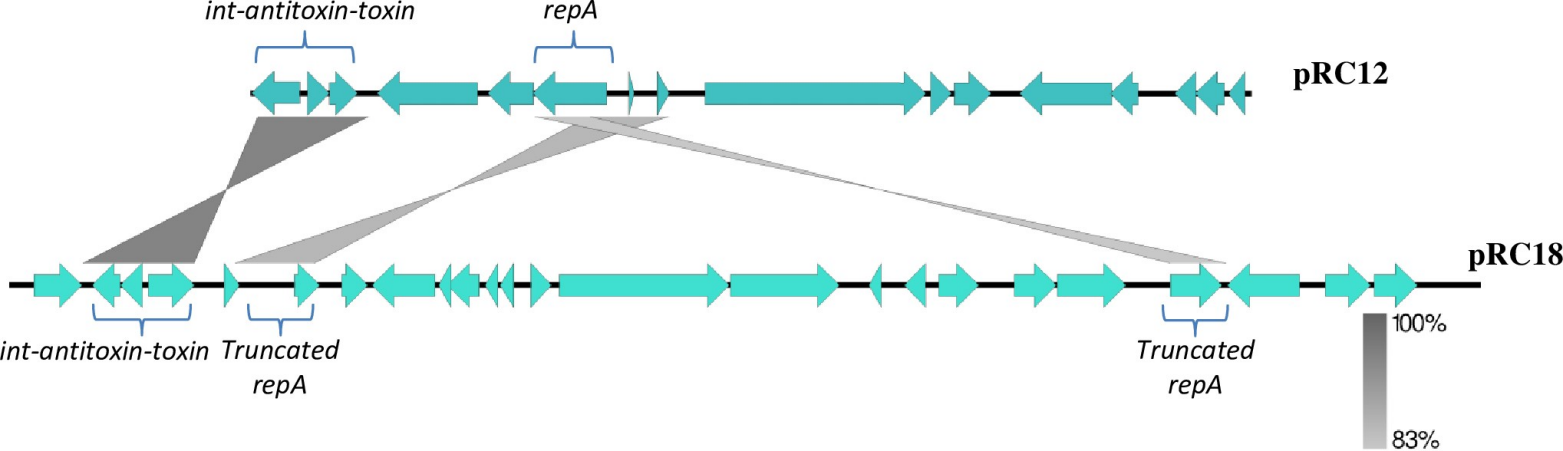
Comparison between pRC12 and pRC18 plasmids. The grey scale indicates the identity % between nucleotide sequences. Integrase, antitoxin/ toxin, and *repA* (pUCL287-like; disrupted in pRC18 by an 11 kb insertion DNA element) genes are common in plasmids pRC12 and pRC18.

**Fig 4:**
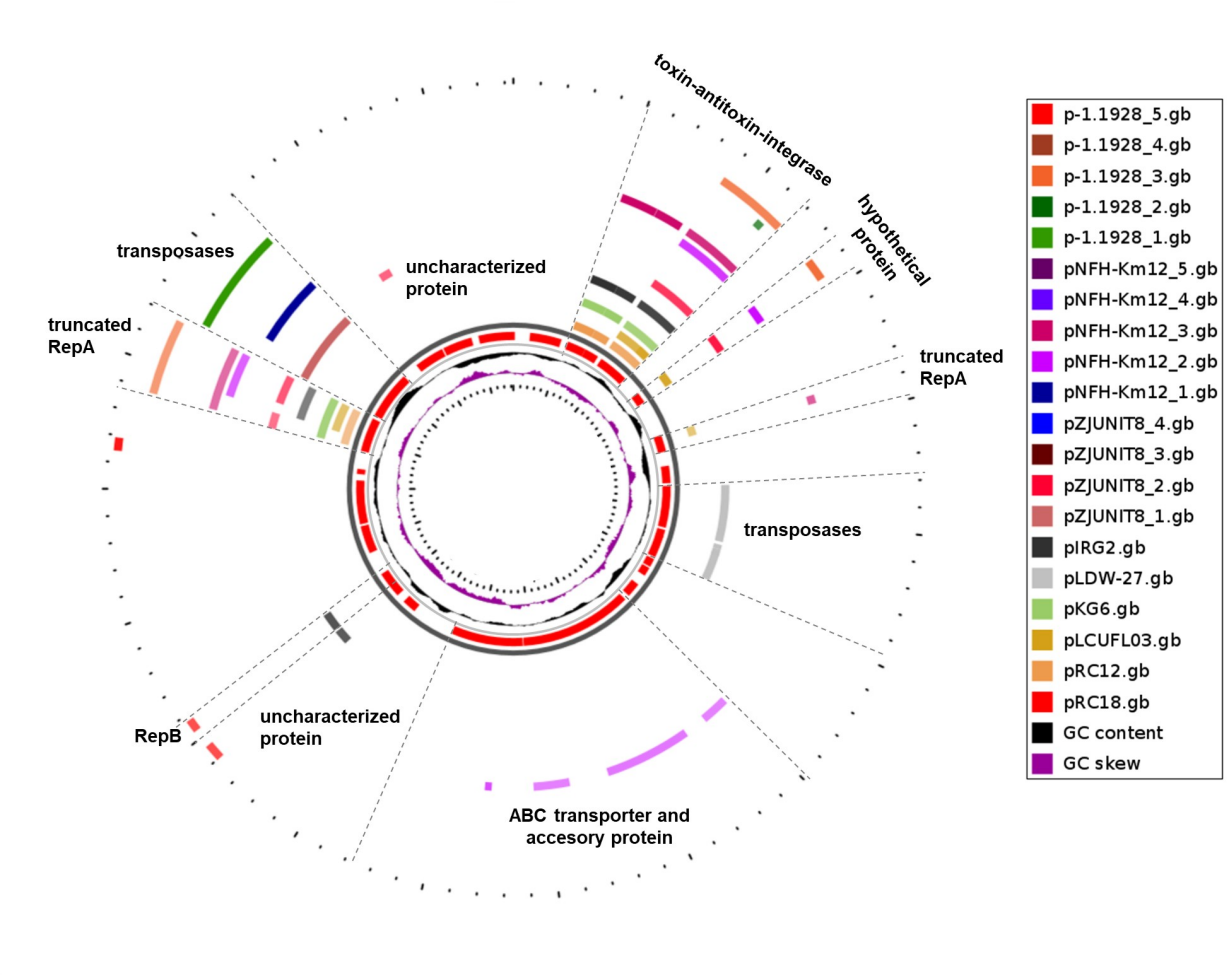
Comparison of pRC18 structural organization with other plasmids. GC skew (purple) and GC content (black) of pRC18 are shown in the inner circles. The pRC18 CDSs (red) are indicated and their paralogs in different plasmids are shown with a color code.

The replication regions, *ori* and *rep* genes, of plasmids pRC12 and pRC18 are not homologous. However, as shown in Fig 3, the *relE*-like toxin- *phd-yefM* type II antitoxin stability systems and the integrase genes of these plasmids shared 100% and 93% identity, respectively. Also, high similarity between the two fragments of the disrupted *repA* in pRC18 and the complete *repA* in pRC12 was found. The toxin-antitoxin system and the integrase were also present (identity score above 80%) in other plasmids of *L*. *curvatus* (see plasmids pKG6, pIRG2, and pZJUNIT8_3; Fig 4) as well as the pUCL287-like RepA protein was found in eight out of the 20 compared plasmids (Fig 4). It is noteworthy that truncated *repA* was only found in pRC18.

### Replication functions of plasmid pRC18. Host range and stability

**Fig 5:**
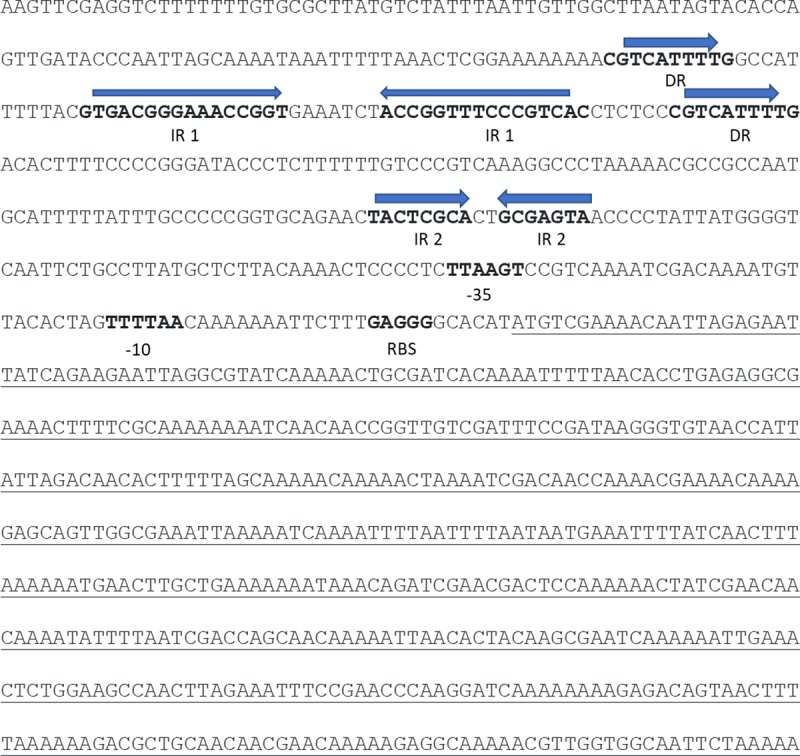
Genetic context of the replication region of the plasmid pRC18. The coding sequence of *repB*’ gene (which encodes a 168 aa protein) is underlined. The sequences of directed repeats (DR) and inverted repeats (IR1 and IR2) are in bold type and indicated with arrows. The predicted position of the -35, -10 boxes and the ribosome binding sites are in bold type.

The replication region of pRC18 is shown in Fig 5. ORF 18 encodes a putative replicase of 168 aa (annotated as RepB’ to differentiate it from the RepB protein of pRC12) that contains a DUF536 superfamily domain (pfam04394) related to theta-replicating plasmids [11, 44]. Moreover, its N-terminus contains a potential HTH domain that might contribute to the interaction with DNA for replication. RepB’ also has 92% identity with a DUF536-containing protein of plasmid pIRG2 from *L*. *curvatus* and 82% of identity with RepB protein of plasmid pLs145-c from *L*. *sakei* LK-145. Upstream of RepB’ (ORF 18), an A-T rich (60%) non-translated region was found with two direct repeats (DR) of 10 bp and two inverted repeats (IR) with potential formation of a stem-loop structure, one of 16 bp at positions 11,518 and 11,541, and the other of 8 bp at positions 11,497 and 11,563 (Fig 5). This region may be the replication origin *ori* of pRC18. Putative promoter regions -35 and -10 are located at positions 11,729 and 11,758, respectively; while a ribosome binding site is predicted at position 11,778, 5 bp upstream from the start codon.

**Fig 6:**
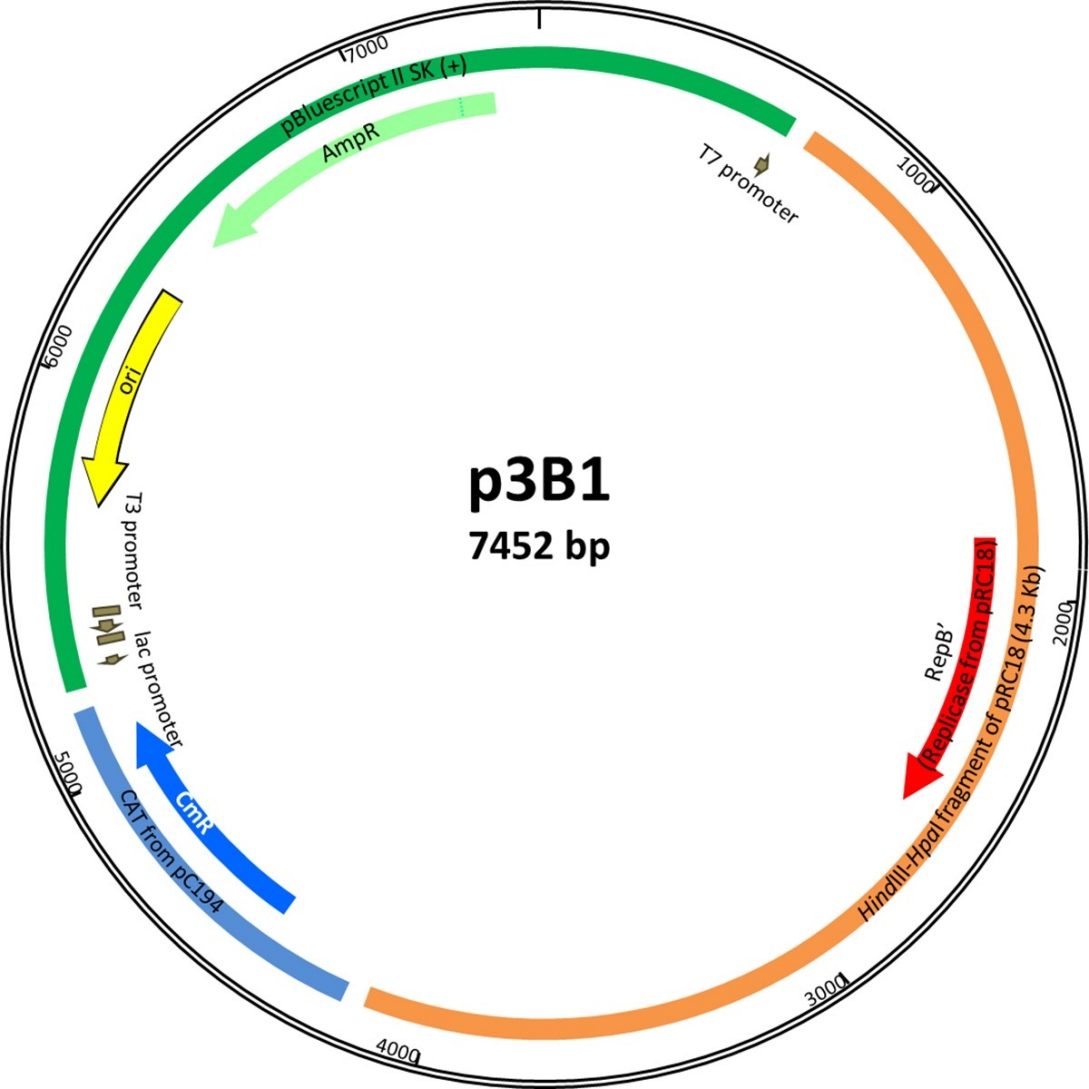
Map of plasmid p3B1. It was constructed by ligating a 3.4 kb *Hpa*I-*EcoR*I fragment of pRC18 (coordinates nt 10334–13806) into the *Hind*III (blunted)-*EcoR*I sites of a derivative-plasmid pBlueScript II SK (+) containing the chloramphenicol (cat) gene from plasmid pC194.

To test the functionality of *ori—*RepB’, a 3.4 kb *HpaI*-*Eco*RI fragment, between coordinates 10,334–13,806 of pRC18, was cloned in a derivative-pBluescript SKII (+) plasmid, which does not have any *ori* for Gram positive bacteria and thus cannot replicate in LAB. This vector contains a chloramphenicol resistance cassette from plasmid pC194 for selection. The recombinant plasmid, named p3B1 (Fig 6), was initially successfully used to transform *L*. *curvatus* CRL 1890, a plasmid-cured derivative of *L*. *curvatus* CRL 705. Transformation efficiency could be improved by optimizing transformation parameters. Indeed the higher efficiencies were obtained using 2.0 kV, 1000 Ω and 2.5 kV, 600 Ω, keeping 25 **μ**F constant. The presence of p3B1 was confirmed by agarose gel electrophoresis and PCR (S3 Fig). Plasmid p3B1 electroporation was also successful in 3 out of 5 tested LAB strains (Table 5), the highest frequency being obtained with *L*. *sakei* 23K with transformation efficiencies higher than those obtained with other plasmids in this strain [9, 11, 14]. This gives new alternatives for the development of genetic tools for this well characterized strain.

Furthermore, we have tested the stability of p3B1 in the absence of selective pressure in *L*. *curvatus* CRL 1890, *L*. *plantarum* CRL 691 and *L*. *sakei* 23K transformants. After approximately 100 generations of cells growing in the absence of antibiotic, the percentages of chloramphenicol resistant colonies were 29±6%, 53±12% and 36±8%, respectively.

**Table 5:** Electrotransformation of p3B1 in different LAB hosts.

Recipient strain	Frequency of transformation p3B1a	Positive control	
		pRV566	pGK12
*L*. *curvatus* CRL 1890	(+)	(46 ± 6) x 10^2^	(12 ± 5) x 10^3^
*L*. *curvatus* DSM 20019	≤1.0 x 10^2^	≤1.0 x 10^2^	ND
*L*. *sakei* 23K	(11 ± 7) x 10^6^	(30 ± 2) x 10^3^	(27 ± 6) x 10^2^
*L*. *plantarum* CRL 691	(7 ± 4) x 10^2^	ND	(+)
*Lactococcus lactis* NZ9000	≤1.0 x 10^2^	ND	(21 ± 3) x 10^3^

^a^ Expressed as the number of antibiotic (according to the plasmid, chloramphenicol or eritromycin) resistant CFU per **μ**g of DNA after electroporation. The transformations were done independently in triplicate. ND stands for not determined, according to the positive control used in each case. (+) indicates successful transformation but the frequency was not calculated.

## Discussion

Theta-type replicating plasmids are classified into six classes according to their dependency on three different components: a plasmid-encoded replication protein (Rep), an *ori* and a DNA polymerase encoded by the host [17]. Plasmid pRC12 belongs to the well-known pUCL287-like family described by Benachour et al. [41], and can be clearly assigned to class A. This replication family is characterized by the presence of 3–5 iterons of 20–22 bp in an AT region located upstream the replicase. The presence of *mobA* and *mobC* functions in pRC12 suggests it may be a mobilizable plasmid; this is consistent with its size (lower than 15 kb), but not with its low copy number [7]. The co-occurrence of both pRC18 and pRC12 in *L*. *curvatus* CRL 705 indicates that the replication systems of both plasmids are compatible. Plasmid-curing experiments showed that pRC12 and pRC18 were very stable at 30°C (plasmid loss was lower than 1%). A functional replicase RepB’ and the presence of an *ori* on pRC18 plasmid are presented here. The replication RepB’ protein of pRC18 (168 aa) has not been previously described; it contains a C-terminal DUF536 domain also found in several bacterial proteins that may be involved in a theta-type replication mechanism. Upstream from the *repB*’ gene, an AT-rich region containing direct and inverted repeats strongly suggest this region could be considered to be the pRC18 replication origin [45]. This structure could act as recognition sites in the initiation of the replication by RepB’ or in the regulation of RepB’ synthesis [46]. The structural organization of the *ori* may differ among plasmids families, e.g. pAMβ1 *ori* consists of a short AT-rich region but does not have a typical structure [47]. In order to classify pRC18 into one of the six classes, further studies addressing the dependency of the *ori* region and the DNA polymerase of the host are necessary.

The genome of *Lactobacillus* has a complex architecture [5], which also applies to its plasmids [9, 48, 49]. While plasmid pRC12 exhibits an average GC content (43.99%) similar to that of the *L*. *curvatus* CRL 705 chromosome (41.95%), the average GC content of pRC18 (34.33%) is lower than that of its host and suggests horizontal genetic transfer. Indeed, pRC18 shows a mosaic structure. The truncated nonfunctional pUCL287-like RepA in pRC18 is explained due to the presence of an 11 kb insertion rich in transposases and *repB*’ functions. pRC12 and pRC18 shared more than 83% nucleotide sequence identity in some regions (toxin/antitoxin, integrase and *repA* genes) located outside the 11 kb insertion, suggesting that this fragment was gained by pRC12 after an integration event into its *repA* gene leading to a second plasmid, pRC18. Although the plasmidome of *L*. *curvatus* is heterogeneous in terms of gene and GC contents [17], some regions like the toxin-antitoxin, integrase and RepA are maintained in other *L*. *curvatus* plasmids such as pGk6, pIRG2 and pZJUNIT8_3.

Plasmids are dispensable extrachromosomal elements. The persistence of plasmids in their bacterial hosts may result from the presence of plasmid genes encoding benefits for the cells to grow under certain environmental conditions [6, 50] or by carrying specialized plasmid stabilization mechanisms such as multimer resolution systems, partition determinants, and post-segregational killing systems (PSK). PSK include toxin-antitoxin systems, restriction modification loci and bacteriocin loci; these systems ensure plasmid maintenance by killing plasmid-free cells [51]. The toxin-antitoxin (TA) systems are a mechanism of plasmid persistence; the antitoxin is more labile and susceptible to degradation than the toxin; thus, in the presence of a plasmid-free cell the toxin induces cell death [52]. Bacterial TA systems are mobile, two-genes modules widely distributed in plasmids and chromosomes, often in multiple copies. TA systems have already been described in lactobacilli [53]; from 1 to 9 TA pair systems have been identified in *Lactobacillus* genomes (https://bioinfo-mml.sjtu.edu.cn/TADB2/browse_org.php?alpha=L). The genetic organization of 6 type II TA systems detected in several *Lactobacillus rhamnosus* and *Lactobacillus fermentum* strains, isolated from various human microbiota, was found to be polymorphic; it was suggested the polymorphisms of the TA systems could be used to the analysis of strains diversity among the *Lactobacillus* genus [54]. The TA systems detected in the plasmids of strain *L*. *curvatus* CRL 705 are type II; both toxins and antitoxins are small proteins and their genes form an operon. Here, RelE-like toxins, which belong to the ParE/RelE superfamily, are combined with antitoxins of different families in the formation of the TA systems. The YefM-RelE-like system is present in both plasmids, pRC12 and pRC18 (100% identity). The YefM antitoxin is from the Phd superfamily. Plasmid pRC12 carries a second TA, the Cro/C1-type HTH domain/HigB-like system [55]. The HigB toxin belongs to the COG4679 family of proteins often found in genomes of phages [55]. The antitoxins to HigB contain a typical HTH domain of the Cro/C1 family and belong to COG1396. Similar to the canonical association is RelB-RelE in *E*. *coli*, the RelE-like toxins may function as a ribosome-dependent mRNA interferase, degrading mRNA positioned at the ribosomal A-site and causing growth arrest and cell death. The antitoxins would bind tightly to RelE inhibiting its ribonuclease activity. The antitoxin could also function as an autorepressor; they can bind alone or in a complex with RelE-like to the operators in the TA promoter blocking its transcription. In *E*. *coli*, TA systems regulate key cellular processes during period of stress; when conditions of stress dissolve, the toxic effect of RelB-RelE is reverse by a *trans-*translation mechanism, allowing growth cells to resume [56]. As indicated above, both plasmids pRC12 and pRC18 were very stable at 30°C (plasmid loss lower than 1%). The stability tests of plasmid p3B1, which is free of the toxin-antitoxin systems, revealed a plasmid loss frequency of >47% plasmid-free cells after 100 generations at 30°C. However, the type II RelE/Phd-YefM system appeared to contribute modestly to plasmid stability of p3B1, since this TA system only slightly improved stability of the recombinant plasmid pRA1TA (plasmid loss frequency of about 65%). These data suggest that the toxin-antitoxin systems present in pRC18 may not contribute by itself to the stability and maintenance of both low copy number plasmids [57]. The presence of the lactocin Lac705 operon, a two-component class IIb bacteriocin [43], in pRC18 could also contribute to the stability of this plasmid by a PSK mechanism.

Results presented here contribute to a better knowledge of *Lactobacillus curvatus* CRL 705, an important strain used as protective cell culture in industrial applications. The small size of RepB**’** (168 aa) from pRC18 represents an advantage for constructing vectors that are easier to handle and likely at high transformation efficiencies. In accordance with its mode of replication, pRC18 derivative vector has a narrow host range, as previously described for theta plasmids [8]. Nevertheless, pRC18 has a replicon that could transform *L*. *curvatus* CRL 1890, *L*. *plantarum* CRL 691 and *L*. *sakei* 23K and be potentially useful to construct cloning vectors for *Lactobacillus*. The vector p3B1 also has suitable unique restriction sites to be used as cloning sites (*Bmt*I, *Nhe*I, *Blp*I, *Bgl*II,*Sac*I, *Bst*XI, *Sac*II, *Ale*I, *Not*I, *Eag*I, *Xba*I and *Bam*HI). Such new cloning vectors for *Lactobacillus* strains will be helpful to study the functionality of different genes and gain knowledge about many functional or probiotic properties of lactobacilli important for food fermentation or biopreservation.

## Supporting information

S1 Fig Nucleotidic comparison of the replication region of plasmids pRC12 from *L*. *curvatus* CRL 705 and pUCL287 from *Tetragenococcus halophilus* ATCC33315. Alignments performed by Clustal Omega shows that plasmid pRC12 has a homologous replicon (*ori*-*repA*) with plasmid pUCL287. The pRC12 plasmid sequence is presented above and the pUCL287 sequence below, the homology is represented by asterisks (*), the numeration in the left is arbitrary in terms of localization in the plasmid.
(PDF)

S2 Fig Comparison between the 11 kb fragment of plasmid pRC18 from *L*. *curvatus* CRL 705 with contig 15 of the whole genome sequence of *L*. *sakei* CECT 9267. Plasmid pRC18 from *L*. *curvatus* CRL 705 is represented in purple while contig 15 of *L*. *sakei* CECT 9267 in red. The identities are highly similar (>99%). The comparison of both sequences was performed using GView Server.
(TIF)

S3 Fig Agarose gel electrophoresis. A) MW: 1 Kb plus DNA ladder (Invitrogen, Carlsbad, CA, USA); Lane 1: p3B1 after plasmid DNA extraction from an electrotransformed colony of *L*. *sakei* 23 K. B) MW: 1 Kb DNA ladder (NEB, Hitchin, UK); Lane 1: PCR fragment that corresponds to RepB of p3B1.
(TIF)
