# Supplementary material for: Nucleotide sequence and analysis of pRC12 and pRC18, two theta-replicating plasmids harbored by Lactobacillus curvatus CRL 705
Source: PLoS One. 2020 Apr 2;15(4):e0230857. doi: 10.1371/journal.pone.0230857 (PMC7117683; doi:10.1371/journal.pone.0230857)
Supplement: S1 Fig — Alignments performed by Clustal Omega shows that plasmid pRC12 has a homologous replicon (ori-repA) with plasmid pUCL287. The pRC12 plasmid sequence is presented above and the pUCL287 sequence below, the homology is represented by asterisks (*), the numeration in the left is arbitrary in terms of localization in the plasmid. (PDF) [file pone.0230857.s001.pdf]

```
pRC12      CCTCTTATATACCTCTTTTATAAACCTCTTTTAAACCTCTTTTAGACCCCTCTTGAGCCT   360
pUCL287    CCTCTTTTATACCTCTTTTATAAACCTCTTTTAAACCTCTTTTAGACCCCTATTGCGCCC   62
           ****** ******************************************** *** ***

pRC12      TACTCTCCTAAGGGTTACAGGACTTTATCGACTACATTTTGTCTGTTTATCGACTACATT   420
pUCL287    TACTCTCCCAAGGGTTACAGGACTTTATCGACTACATTTTGTCTGTTTATCGACTACATT   122
           ******** ***************************************************

pRC12      TTGTCTGTTTATCGACTACATTTTGTCTGTTTATCGACTACATTTTGTCTGTTTATCGAC   480
pUCL287    TTGTCTGTTTATCGACTACATTTTGTCTGTTTATCGACTACATTTTGTCTGTTTATTGAC   182
           ******************************************************** ***

pRC12      TACACTTATTTACTTCTGTATTCAAATAAAGTAGTATTATTCAAGGAGGTTATTTTATGA   540
pUCL287    TACACTTATTTACTTCTGTATTCAAATAAAGTAGTATTATTCAAGGAGGTTATTTTATGA   242
           ************************************************************

pRC12      GCAATGAATTAGTTAAATATGATCCAGAACTGAATACAATTCCTCTGAGAAGATTTACTC   600
pUCL287    GCAATGAATTAGTTAAATATGATCCAGAACTGAATACAATTCCTCTGAGAAGATTTACTC   302
           ************************************************************

pRC12      CTGTAGAAATGAATCTTTTCTTTTCGGTTGTTTCTAGAATGCGCGATAAAGGTGATGATA   660
pUCL287    CTGTAGAAATGAATCTTTTCTTTTCGGTTGTTTCTAGAATGCGCGATAAAGGTGATGATA   362
           ************************************************************

pRC12      CTGTTCGGTTTACTTTTGATCAGTTAAAAGAATTAAGCGCGTATAAGCCGACCGCAAATA   720
pUCL287    CTGTTCGGTTTACTTTTGATCAGTTAAAAGAATTAAGCGCGTATAAGCCGACCGCAAATA   422
           ************************************************************

pRC12      ATCGATTTATTGATGACATACAAAGTACATATCAAAAAATATTAGGTCTTAGATTTGGCT   780
pUCL287    ATCGATTTATTGATGACATACAAAGTACATATCAAAAAATATTAGGTCTTAGATTTGGCT   482
           ************************************************************

pRC12      CTAGAAGTAAAGATGGACTTGATAGAGAAATGTTTGTCATGTTTACTCGATTTGAAATCA   840
pUCL287    CTAGAAGTAAAGATGGACTTGATAGAGAAATGTTTGTCATGTTTACTCGATTTGAAATCA   542
           ************************************************************

pRC12      AGGGTTCGGCAGAAGTCCCTTATGTTGATATTCAAATTTACCCCAAGGCATTGAAACTTC   900
pUCL287    AGGGTTCGGCAGAAGTCCCTTATGTTGATATTCAAATTTACCCCAAGGCATTGAAACTTC   602
           ************************************************************
```

The four direct repeats of 11-bp (5´-CCTCTTTTATA-3´) are shown in blue; the second and fourth repeats are underlined. Two adenines (in bold) are located in the middle of these repeats.

A mismatch of 4 bases is shown in the conserved 37-bp nucleotide sequence (in magenta, bold letters) between both plasmids.

The 4.5-times iterons (5’-TTTATCGACTACATTTTGTCTG-3´) are indicated in red with intercalated underlining.

The region where promotor and ribosome binding sites are located is represented with black bold letters. *repA* gene (936-bp) is highlighted in yellow. Overlapping (8-bp) between *repA* and *repB* is indicated by double.

repA gene (936-bp) is highlighted in yellow.

```
pRC12      TAAATAATCTTGAAAGCTGGGTTCGATATGCTTTAGCAGAGTTCAGAGATTTAAAGAGTA   960
pUCL287    TAAATAATCTTGAAAGCTGGGTTCGATATGCTTTAGCAGAGTTCAGAGATTTAAAGAGTA   662
           ************************************************************

pRC12      GTTATGCAAAAACGATGTTTCGTCTCCTTAAACAATTTCGAACTACTGGTTATGCTTATT   1020
pUCL287    GTTATGCAAAAACGATGTTTCGTCTCCTTAAACAATTTCGAACTACTGGTTATGCTTATT   722
           ************************************************************

pRC12      TTTCTAAAAGTGATTTTTTTGAATTACTTGATATTCCGCAAAGCTATTGGAATAAACCTG   1080
pUCL287    TTTCTAAAAGTGATTTTTTTGAATTACTTGATATTCCGCAAAGCTATTGGAATAAACCTG   782
           ************************************************************

pRC12      CAAACGTTGAATCCAGAGTTATTCAGCCAATTAGAGAAGAATTAACCCCGCTTTTTAGAG   1140
pUCL287    CAAACGTTGAATCCAGAGTTATTCAGCCAATTAGAGAAGAATTAACCCCGCTTTTTAGAG   842
           ************************************************************

pRC12      GGCTAACGATTAGAAAAAAATATGGTAAAGGCCGTGGCAAGCCGGTTATTGGTTACTCAT   1200
pUCL287    GGCTAACGATTAGAAAAAAATATGGTAAAGGCCGTGGCAAGCCGGTTATTGGTTACTCAT   902
           ************************************************************

pRC12      TTACCTGGAAATCAGAAAAAAAAGAACGCAGACGACTTCTCACAAGGGCAATTTCAAGATG   1260
pUCL287    TTACCTGGAAATCAGAAAAAAAAGAACGCAGACGACTTCTCACAAGGGCAATTTCAAGATG   962
           ************************************************************

pRC12      AAAGACAAAAGCTTTTCAATATTCAGCATAATAGTGAACTAACAGAACATCAAAAATGGC   1320
pUCL287    AAAGACAAAAGCTTTTCAATATTCAGCATAATGGTGAATTAACAGAACAGGAAAAATGGC   1022
           ******************************** ***** **********  *********

pRC12      GCGCCATTGACAAGGTTAAGGGGTTAACTTTAGGCTCTACTGAGAAACAAGCATTGGCTG   1380
pUCL287    GCGCCATTGATAAAGTTAAGGAGTTAACTCTAGGCTCTACTGAGAAACAAGCATTGGCTG   1082
           ********** ** ******* ******* ******************************

pRC12      ATAAACAGGCCGAGCACGATAAAAAAATAAGAGATCAAGCAAGACAAGAAGCACTTGCTG   1440
pUCL287    ATAAACAGGCCGAGCACGATAAAAAAATAAGAGATCAAGCAAGAAAAGAAACACTTGCTG   1142
           ******************************************** ***** *********

pRC12      AACTCCGAAAGGGGTTTGGAAATAATGCCTAAAACAATTAGAGAACTTGCTGACGAATTG   1500
pUCL287    AACTCCGAAAGGGGTTTGGAAATCATGCCTAAAACTATTAGAGAACTTGCTGATGAATTG   1202
           *********************** *********** ***************** ******
```

Overlapping (8-bp) between *repA* and *repB* is indicated by double underlining.

```
pRC12         AAGGTCTCTAAACAAACTATTCAATACCACTACCAAAGACTACCAACAAAGAACCGACAA    1560
pUCL287       GGCGTTTCAAAGCAAAGAATTCAACAAATTATCGCCAAATTATCGCCAAGCAAAACGCCA    1262
                 ** ** ** ****  ****** *      *   * * ** *  ***  **    * *

pRC12         AAAGATAGTCAAGGTACAAACATGATCAGCCTTACAGCTGAAAGGATTATTAGGGACAAG    1620
pUCL287       AATAAGGAAGGCAATAGATATGTTTTGAGCGCCCAAGATGTCAAAAATATAAAGGCTT--    1320
              **  *         ** * *  *  * ***     ** **  *  * *** * **

pRC12         GTAGCAAAGCCTTTGGTAGCAAATACCCAACAAACAG---GTAGCAAAAAAGTGACAAAG    1677
pUCL287       ---TGATGGGATTTGAAAATAACAAGTCATCAACAAGTGAATCGACAAATAGACTTGTTG    1377
                   *  *  ****  *  **  *  ** ***  **    * *  *** **       *

pRC12         ACTAGCAAAGAAAATAATGAGCTAATTGCCACTCTAAGAAGAGAAATAGAAGATTTAAAG    1737
pUCL287       ATTATGATGT-TTACTTAGATGTGATAGATTCTATAAAAGAAAA------AGATGAACAG    1430
              * **  *      *    **  * ** *   ** *** *  * *      ****  * **

pRC12         TCTCAACGTAACAAACAGCTTGCTACCAAAGACCGACAAATAGATCATCTAACAAAATTG    1797
pUCL287       ATAAAAAGTTTATTAGAA------------------GTTCAAAAACAAACACAAAATTTA    1472
                  ** **     * *                         * * **   *  *** **

pRC12         GTGGATCAGCAGCAACAATTACAATTAGCAACAGTAGCAGATAACCGTCGATTAAAAGAT    1857
pUCL287       TTAGATCAGCAGCAACGATTAGCATTACAGGATAAAAAACTTTTAGAA-GAATACAAG--    1529
               * ************* ****  ****        *  *  *       ** ** ***

pRC12         CATGTACAAAAGCTAAGTGGGCAACTAACTCAAAAAACTAACGACAACTTGTCGACCGGA    1917
pUCL287       ----ACAGAAATCAAAGAATTAAAATCATTAAATATACCAAAACAAGGTAGTGAAA-AAG    1584
                      *** * ***     ** * * * ** * ** **    *  * **  *

pRC12         AATGATCTTTTTAACATCCAAGATA-------AAAAAAGCAAAATAGCTA---AACAGAA    1967
pUCL287       ACGATTCTATTCCAAAAGAAAATTCAGCAGAAAATAGAGTAAAAGAGCCTCAAAATAAAA    1644
              *    *** **  * *   **  *        ** * ** **** ***     ** * **

pRC12         GAT-TGCTAAATCTGGTAGTAATAAAGATGGCATACACACAAATAGAGCTATTAAACGTT    2026
pUCL287       AATGGTGGCACTTTGGTAGGAGAATGTAATGAAGACACAAACTTTCAATGATTTAATTGA    1704
               **      * * ****** *  *   *  * * ***** *  *  *   *** **

pRC12         GGTGGAAATTCTGGTAAAAGTTAATGTAAGCCTTAAGGTTTCAACTAAAGCAATTTACGA    2086
pUCL287       GCAAATTAATCAGGCTGCAGGTGATGTCCAACGTGAA-----------------------    1741
              *      * ** **    ** * ****    * * *
```

repB from pRC12 (579-bp) and from pUCL287 (507-bp) are highlighted in grey.

The aligment between sequences of plasmids pRC12 and pUCL287 were performed using Clustal Omega.

Sievers F, Wilm A, Dineen D, Gibson TJ, Karplus K, Li W, et al. Fast, scalable generation of high-quality protein multiple sequence alignments using Clustal Omega. Mol Syst Biol. 2011; 7:539. doi: 10.1038/msb.2011.75
