# Supplementary figures and images for: Nucleotide sequence and analysis of pRC12 and pRC18, two theta-replicating plasmids harbored by Lactobacillus curvatus CRL 705
Source: PLoS One. 2020 Apr 2;15(4):e0230857. doi: 10.1371/journal.pone.0230857 (PMC7117683; doi:10.1371/journal.pone.0230857)

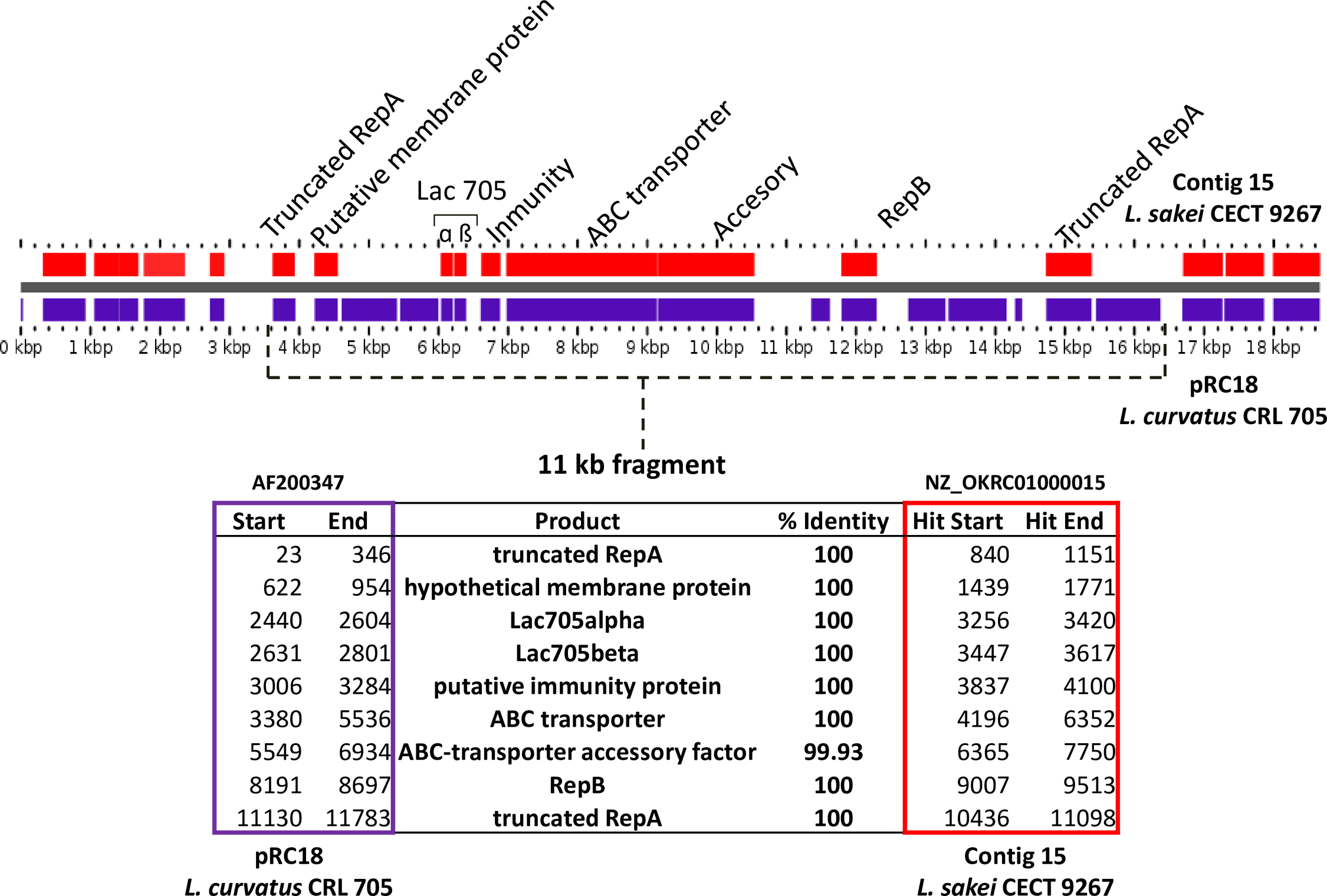

Supplement: S2 Fig — Plasmid pRC18 from L. curvatus CRL 705 is represented in purple while contig 15 of L. sakei CECT 9267 in red. The identities are highly similar (>99%). The comparison of both sequences was performed using GView Server. (TIF) [file pone.0230857.s002.tif]

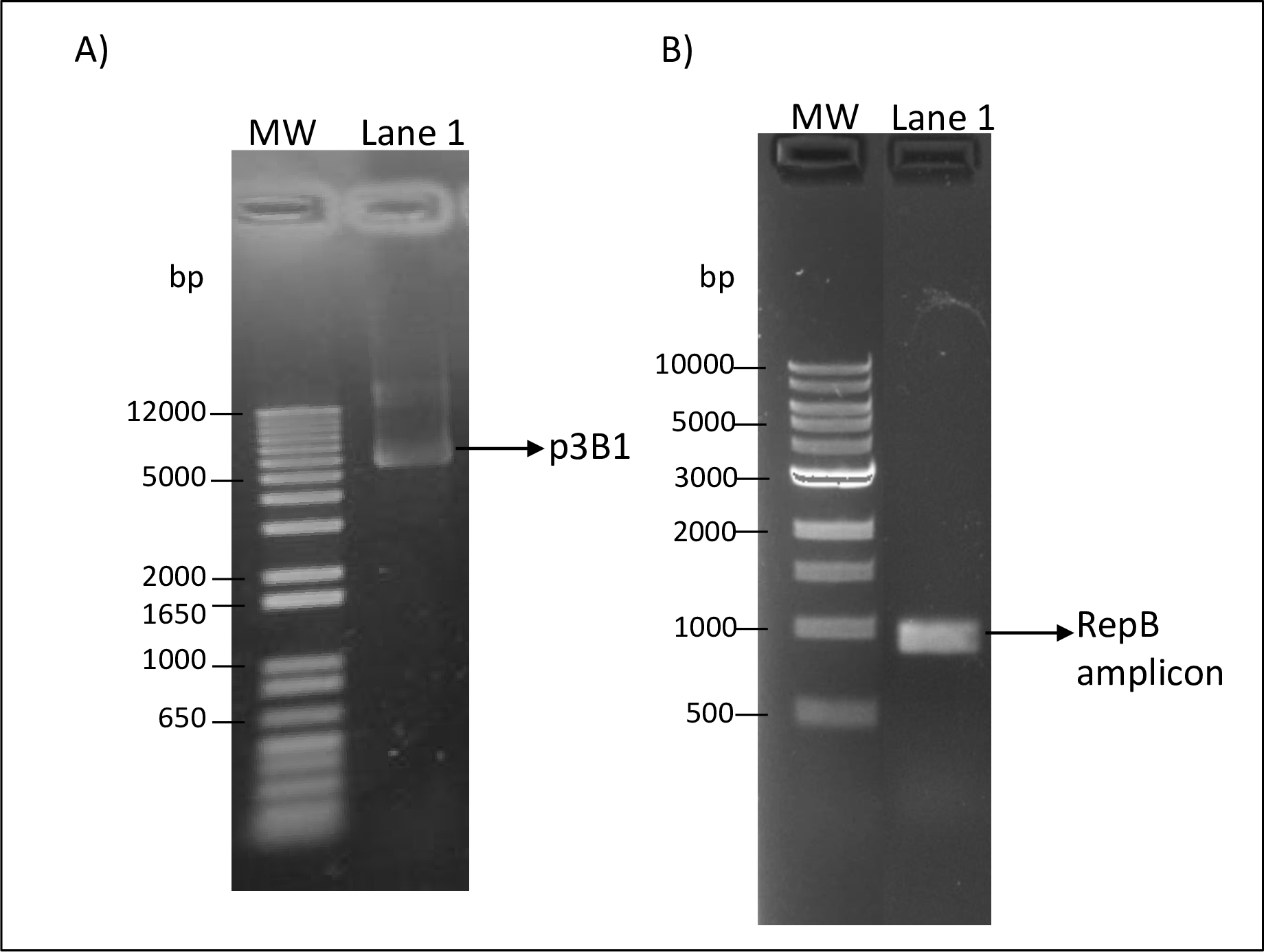

Supplement: S3 Fig — A) MW: 1 Kb plus DNA ladder (Invitrogen, Carlsbad, CA, USA); Lane 1: p3B1 after plasmid DNA extraction from an electrotransformed colony of L. sakei 23 K. B) MW: 1 Kb DNA ladder (NEB, Hitchin, UK); Lane 1: PCR fragment that corresponds to RepB of p3B1. (TIF) [file pone.0230857.s003.tif]
